# 943. Epidemiology of Actinomycosis in a Tertiary Care Cancer Center

**DOI:** 10.1093/ofid/ofab466.1138

**Published:** 2021-12-04

**Authors:** Mohammad El-Atoum, Nikolaos Almyroudis, Katherine M Mullin, Brahm H Segal

**Affiliations:** 1 University at Buffalo, Lancaster, New York; 2 Roswell Park Comprehensive Cancer Center, Buffalo, NY, Buffalo, New York

## Abstract

**Background:**

Actinomyces are human commensals with significant pathogenic potential. The aim of this study was to determine the epidemiology of Actinomycosis in a tertiary care cancer center and identify species most commonly associated with invasive disease.

**Methods:**

We retrospectively reviewed all patients referred to our institution with suspected or documented solid or hematological malignancies and positive cultures for Actinomyces species from July 2007 to June 2020 (13 years). Species identification was performed by VITEK^®^ automated system (bioMerieux Inc.). Probable invasive actinomycosis was defined as cases with consistent clinical presentation, suggestive radiographic findings, and a positive culture from a nonsterile site, but lack of histopathological confirmation. Proven invasive actinomycosis was defined as the presence of consistent clinical symptoms, suggestive radiographic findings, a positive culture and histopathological confirmation, or cultures from sterile site without histopathological confirmation. Contaminants were considered positive cultures from sterile or non-sterile site without evidence of disease.

**Results:**

Of 233 cases with positive cultures 194 (83.3%) were considered contaminants and 39 (16.7%) diagnostic of invasive actinomycosis. Of 39 cases of invasive actinomycosis, 64% were documented in patients with solid tumors, 13% in hematological malignancy and 23% among individuals without proven malignancy, 25 (64%) were probable and 14 (36%) proven. Of patients with proven/probable actinomycosis 27 (69%) had polymicrobial growth. Abdominopelvic was the most frequent site of invasive actinomycosis. A. odontolyticus was the most common species isolated (41%) followed by A. meyeri (28%) in patients with invasive disease, and A. odontolyticus (42%) among contaminants.

**Conclusion:**

The majority of positive cultures for Actinomyces species were considered contaminants. In our cohort Invasive actinomycosis affected mainly patients with solid tumors. Abdominopelvic was the most common site of invasive disease. Species most commonly associated with invasive actinomycosis were A. odontolyticus followed by A. meyeri with A. israelii isolated less frequently.

**Disclosures:**

**All Authors**: No reported disclosures

